# Immunohistochemical dynamics of cell wall matrix polymers during tomato autograft healing

**DOI:** 10.1007/s11103-023-01351-7

**Published:** 2023-04-20

**Authors:** Carlos Frey, Nerea Martínez-Romera, Antonio Encina, José L. Acebes

**Affiliations:** Área de Fisiología Vegetal, Departamento de Ingeniería y Ciencias Agrarias, Facultad de Ciencias Biológicas y Ambientales, Universidad de León, Campus Vegazana, 24007 León, Spain

**Keywords:** Arabinogalactan proteins, Grafting, Immunohistochemistry, Pectin, Tomato (*Solanum lycopersicum*), Xyloglucan

## Abstract

A large part of the production of tomato plants is grafted. Although it has recently been described that cell walls play an important role in tomato graft healing, the spatiotemporal dynamics of cell wall changes in this critical process remains largely unknown. The aim of this work was to immunolocalize changes in the major cell wall matrix components of autograft union tissues throughout the course of healing, from 1 to 20 days after grafting (DAG). Homogalacturonan was de novo synthetized and deposited in the cut edges, displaying the low methyl-esterified homogalacturonan a stronger labelling. Labelling of galactan side chains of rhamnogalacturonan increased until 8 DAG, although remarkably a set of cells at the graft union did not show labelling for this epitope. Changes in xylan immunolocalization were associated to the xylem vasculature development throughout, while those of xyloglucan revealed early synthesis at the cut edges. Arabinogalactan proteins increased up to 8 DAG and showed scion-rootstock asymmetry, with a higher extent in the scion. The combination of these changes appears to be related with the success of the autograft, specifically facilitating the adhesion phase between scion-rootstock tissues. This knowledge paves the way for improved grafting using methods that facilitate appropriate changes in the time and space dynamics of these cell wall compounds.

**Supplementary Information:**

The online version contains supplementary material available at 10.1007/s11103-023-01351-7.

## Key message

Immunolocalization reveals that spatiotemporal changes of cell wall components of the graft union tissue, such as homogalacturonan, xyloglucan and arabinogalactan proteins, have an important role for successful graft healing in tomato.

**Supplementary Information:**

The online version contains supplementary material available at 10.1007/s11103-023-01351-7.

## Introduction

Plant grafting is an ancient horticultural practice that consists of joining different parts of plants. Generally, a graft is a combination of two parts: the rootstock (includes root system) and the scion (includes the aerial part). For the graft to be successful, a complex set of morphogenetic and developmental processes is required. These processes involve physiological, molecular and gene expression changes at the graft junction, leading, first, to adhesion between the rootstock and scion tissues and, second, to vascular reconnection between both partners (Melnyk 2017).

Although plant grafting has been used for centuries, nowadays it has acquired a huge economic impact for the agricultural industry, being routinely used in some of the most valuable crops such as vines, fruit trees (citrus), cucurbitaceous (melon) and solanaceous (tomato) vegetable crops (Lee et al. 2010).

Tomato cultivation is a very important horticultural crop worldwide. In 2021, 189,134 million tons of tomatoes were produced worldwide, on more than 5 million hectares (FAOSTAT 2022). The relevance of this crop suggests that any improvement in its yield would generate a large economic impact. Nowadays, tomato grafting is used to increase tomato vigour and disease resistance by utilizing special rootstocks (Singh et al. 2017; Grieneisen et al. 2018).

Changes occurring in the rootstock and scion tissues during graft formation can be grouped into three stages (Melnyk 2017):

o An initial tissue damage response, followed by cell adhesion and callus formation at the graft junction zone. A necrotic layer appears, and callus is formed as a consequence of cell de-differentiation and proliferation (Jeffree and Yeoman 1983; Pitaksaringkarn et al. 2014; Sala et al. 2019).
o Tissue cohesion and vascular cells differentiation. Necrotic layer becomes disorganized, new shared cell wall is developed between scion and rootstock junction cells, and certain groups of cells within the callus are determined to vascular meristems that become eventually differentiated in new conducting elements (Jeffree and Yeoman 1983; Wang and Kollmann 1996; Pina et al. 2012; Melnyk et al. 2015).
o Vascular reconnection. Finally, *de-novo* vascular tissues contact within the graft zone and vascular integrity of stem is restored by the differentiation of a continuous vascular system between scion and rootstock (Melnyk et al. 2015).

Cell walls provide essential structural and physiological functions in cell–cell recognition, cell shape differentiation and tissue adhesion (Zhang et al. 2021). They are semirigid, dynamic, macromolecular composites that accumulates in the outer face of plasma membranes. Their composition depends greatly on the species and even the cell types and cell developmental stage.

The growing cells are encased by a primary cell wall, consisting of a framework of cellulose microfibrils embedded in a matrix of hemicelulloses, pectins and glycoproteins (e.g. arabinogalactan proteins (AGPs) (Zhang et al. 2021). In tomato stems, as well as in other dicot, the major matrix cell wall polysaccharides are xylan and xyloglucan, among the hemicelluloses, and homogalacturonan (HG) and rhamnogalacturonan I (RGI) and II, among the pectins (Zhang et al. 2021; Frey et al. 2022). When certain cell types stop growing they accumulate a secondary cell wall composed basically by cellulose, xylans and lignin (Zhong et al. 2019).

It is considered that cell walls have an important role in grafting process (Miller and Barnett 1993; Yeoman 1993; Pitaksaringkarn et al. 2014; Melnyk 2017; Frey et al. 2021, 2022). In fact, transcriptomic analysis in tomato and other species has shown that significant overexpression of genes for cell wall biosynthesis and remodelling occurs early after grafting onset (Cookson et al. 2013; Cui et al. 2021; Xie et al. 2022). Cell walls undergo modifications throughout graft healing, having a key role in graft success. First, pectins are actively secreted at the cut edges (Sala et al. 2019; Frey et al. 2020); then new shared cell walls are formed between scion and rootstock and plasmodesmata appear (Jeffree and Yeoman 1983; Pina et al. 2012). Finally, changes in the cell wall of the vascular tissues are required during the vascular reconnection (Melnyk et al. 2015). Although quantitative changes in cell wall composition thorough tomato grafting have been recently reported (Frey et al. 2022), detailed information on the location of those changes in the cell walls of the different cell types, and their progression over time is lacking. This information is relevant to understanding the key factors that explain the formation of a successful graft.

The aim of our work was to localize changes in cell wall composition and structure at the graft interface that putatively occur throughout the whole grafting process (1 to 20 days after grafting, DAG) and that are involved in graft healing in tomato compatible autografts. For this purpose, an immunohistochemistry (IHC) study was carried out for a wide range of cell wall epitopes.

## Materials and methods

### Plant material

Seeds of tomato (*Solanum lycopersicum*) “Minibel” (Mascarell Semillas S.L.) were germinated and grown in containers with 180 mL of black peat-based substrate. After seeds germination they were placed in a growth chamber at 23 ± 1 °C under light (≈ 41 µmol m^−2^ s^−1^) with 16/8 photoperiodic conditions and 50–60% of humidity (Frey et al. 2022). Every three days the plants were watered with complete Hoagland solution rising ≈ 90% of field capacity.

### Autografting method

After approximately five weeks, the stems of the plants reached a diameter of 4–5 mm, and autografts (self-grafting) were performed. A sliding cut (≈ 45°) was made 0.5–1 cm under cotyledonary leaves, and the union between scion and rootstock was assured by a graft clip (Toogoo®). After grafting humidity was maintained at ≈ 90–100% during the first days and progressively reduced at room humidity.

Autografted plants were collected at different times, from 1 to 20 DAG. Three grafts (from scion and rootstock, or from complete graft union beginning at 8 DAG) were taken and processed in order to obtain IHC images.

### Immunolocalization of cell wall components

Stem pieces (≈ 3 × 2 mm) from the rootstocks, scions or autograft junctions were fixed in 2.5% (w/v) paraformaldehyde in 0.1 M phosphate buffer (PBS) pH 7.5 at 4 ℃ overnight (Frey et al. 2021). Fixed samples were dehydrated in a decreasing ethanol series and embedded in resin (LR White, London Resin, Reading, UK). The embedded samples were placed in gelatin capsules with resin and then incubated at 37 °C for 5 days to polymerize the resin. An Ultracut-Microtome LKB 2088 (Reichart-Jung®, Austria) was used to obtain 1 µm thick sections (Fig. S1). These sections were placed on slides coated with Vectabond™ reagent (Vector Laboratories®, Burlingame, CA, USA) and then incubated with M-PBS (Milk-Phosphate Buffered Saline) containing the primary antibody (Plant Probes, Leeds, UK) (Table 1) at a 1/10 dilution for 2 h (García-Angulo et al. 2006). After washing with PBS, the sections were incubated for 2 h with a 1/100 dilution of an anti-rat immunoglobulin G-linked to fluorescein isothiocyanate (Sigma®) in M-PBS. The antibody incubations were performed in darkness and at room temperature. Finally, a contrast staining was performed using 0.005% calcofluor White (fluorescent brightener 28, Sigma®). A Nikon E600 epifluorescence microscope with the UV-2 and B-H2 filters was used to study the immunolabelling *in muro*. Image acquisition software used was Nis-Elements F. v 3.2.

**Table 1:** Monoclonal antibodies used as primary antibodies for IHC

Antibody	Epitope	References
*Pectins: homogalacturonan (HG) and rhamnogalacturonan I (RGI)*		
JIM5	Partially methyl-esterified HG and can also bind to unesterified HG	Knox et al. (1990)
JIM7	Partially methyl-esterified HG but not unesterified HG	Knox et al. (1990)
LM19	Range of HG samples, preference to unesterified HG	Verhertbruggen et al. (2009)
LM20	Does not bind to unesterified HG (requires methyl-esters for recognition)	Verhertbruggen et al. (2009)
LM5	Linear tetrasaccharide in (1–4)-β-d-galactans (RGI side chain)	Jones et al. (1997)
LM6	Linear pentasaccharide in (1–5)-α-l-arabinans (RGI side chain)	Willats et al. (1998)
*Hemicelluloses*		
LM10	Unsubstituted and relatively low-substituted xylans	McCartney et al. (2005)
LM15	XXXG motif of xyloglucan	Marcus et al. (2008)
*Arabinogalactan proteins (AGPs)*		
LM2	AGP (carbohydrate epitope containing β-linked glucuronic acid)	Smallwood et al. (1996)

For immunolocalization using LM19 and LM20 antibodies, the stem pieces were fixed in formaldehyde–acetic acid–alcohol (70% ethanol) (FAA) 48 h at 4 ℃ and dehydrated in a decreasing ethanol series. The pieces were then incubated with isoamyl acetate in an increasing ethanol series prior to paraffin imbibition (Paraplast®). Paraffin blocks were cut on a rotary microtome (LEITZ 1512) to obtain 6 µm thick sections. IHC was performed on these sections in the same manner as described in the previous paragraph, using LM19 and LM20 as primary antibodies.

### Ruthenium red staining

Stem pieces (1 cm of height) from the graft junction were fixed in FAA, imbibed in paraffin, and cut on a microtome as indicated in the previous section.

After deparaffinization, the sections were rehydrated, and the slides were incubated for 30 min with 0.02% (w/v) ruthenium red dissolved in distilled water; the sections were then washed with distilled water for 5 min and observed under a Nikon E600 brightfield microscope.

### Fluorescence quantification

In order to quantify fluorescence emission, images were processed with ImageJ 1.53 k software. Comparison between the fluorescence emission of cell wall structures in the cut edges and internal tissues were evaluated sampling all pixels in a random segment line that passed inside cell wall structures in both zones. These paths crossed the images from one side to the other. Three technical replicates were obtained for each image. The fluorescence (relative units) of the sequence of all pixels was measured and expressed as mean ± SD. The grey value of the pixels was used to determine the fluorescence intensity, ranged from 0 to 255.

## Results

### Pectins

**Fig. 1:**
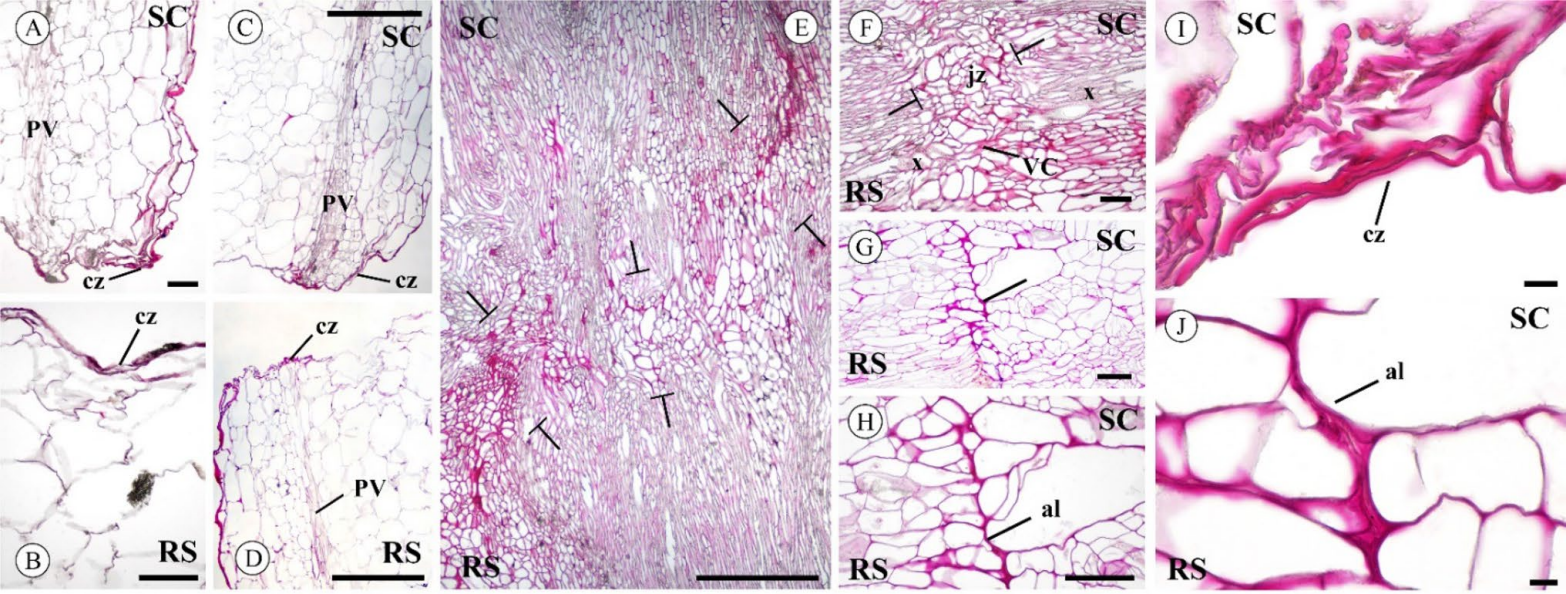
Representative images of tomato autograft longitudinal sections stained by ruthenium red at 1 (**A**, **B**), 2 (**C**, **D**, **I**) and 20 (**E**–**H**, **J**) days after grafting (DAG). At 1 and 2 DAG the union between scion and rootstock is not yet established. **A**–**D** Note the accumulation of pectins in the cut zone (cz) both in the scion and rootstock. **E**–**H** This accumulation of ruthenium red stained pectins remains in the junction zone (jz) at 20 DAG. **E** The junction zone is delineated by T lines (┬). **F**–**H** The adhesion line (al) is usually well visible due to a wide cell wall rich in pectins. **H** is a deeper detail of **G**. **I** Detail at 2 DAG in the cut zone (cz). ** J** Ruthenium red staining is conspicuous at 20 DAG in the adhesion line (al). *Bars*: **A**, **B**, **F**, **G**, **H** = 100 μm. **C**, **D**, **E** = 500 μm. **I**, **J** = 10 μm. *Abbreviations*: *al* adhesion line, *cz* cut zone, *jz* junction zone, *PV* pre-existing vascular tissue, *RS* rootstock, *SC* scion, *VC* vascular connection, *x* xylem. Original source of images: Martínez-Romera et al. (2021)

Pectin accumulation was observed by ruthenium red staining in the cut zone, both in the scion and rootstock as early as 1 and 2 DAG (Fig. 1A–D and I for details). At 20 DAG, a large deposition of pectins was observed, allowing clear identification of the junction zone (Fig. 1E–H and J for details). Pectin deposition sometimes affected several cell layers. Once the union between the scion and the rootstock was established, vascular continuity was detected. Minor staining was observed in the xylem vessels -easily recognizable by the reticulate pattern of their cell walls- compared to the parenchyma and callus cells at the scion-rootstock junction zone, whose cell walls were strongly stained with ruthenium red (Fig. 1E, F).

**Fig. 2:**
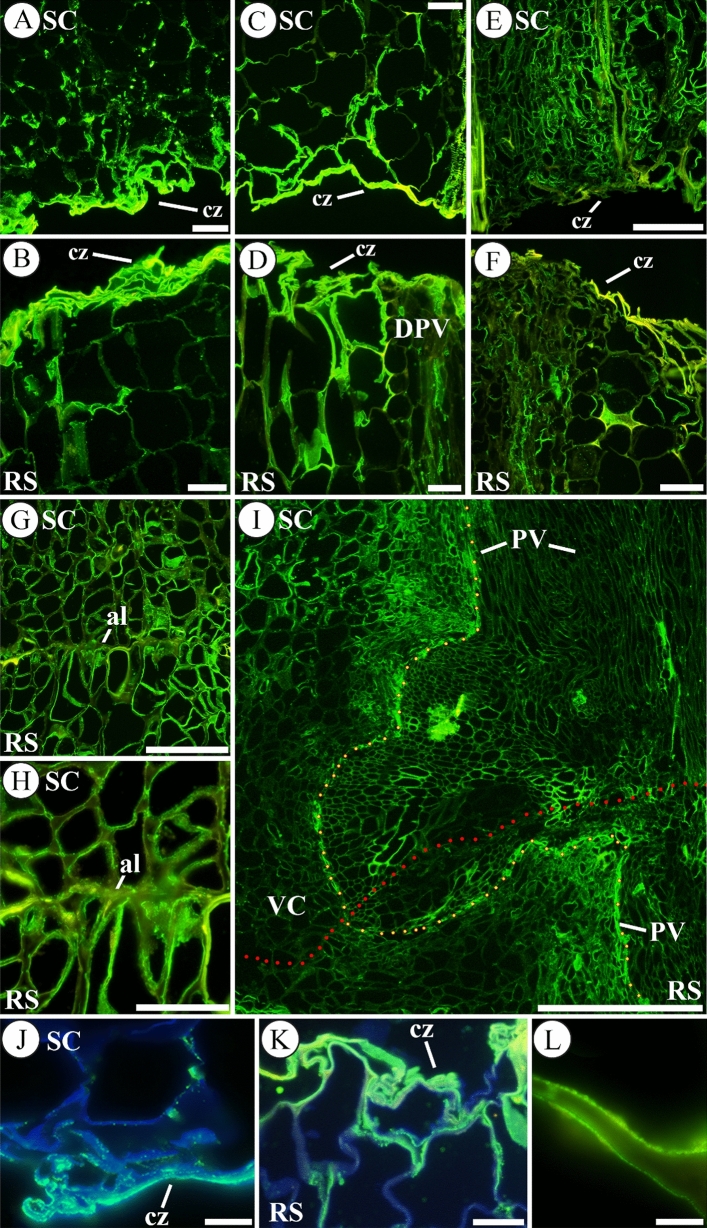
Unesterified HG distribution throughout graft union. Representative images of autograft longitudinal sections immunolabelled with LM19 at 1 (**A**, **B**, **J**), 2 (**C**, **D**, **K**), 4 (**E**, **F**) and 20 (**G**–**I**, **L**) days after grafting (DAG). (**A-D**) At 1, 2 and 4 DAG the union between scion and rootstock is not yet established. Note the accumulation of unesterified HG in the cut zone (cz), even observe this accumulation in the cut zone of a damaged pre-existing vascular tissue (DPV) in **D**. **F**, **G** Proliferation of callus and vascular cell elements was observed near to the cut zone. **G**, **H** Accumulation of unesterified HG in the adhesion line (al) between scion (SC) and rootstock (RS), note that **H** is a detail of **G**. **I** Observe the pre-existing vascular tissue (PV) of the scion and the rootstock reconnection (VC); unesterified HG labelling is widely present in the vasculature. Dotted red line indicates the junction zone and the yellow dotted line traces the pre-existing (PV) and regenerated connecting vasculature (VC) connecting scion and rootstock. **J**, **K** Details of the presence of unesterified HG in the cell walls of the cut zone, even forming a matrix, at 1 and 2 DAG respectively. **L** Detail of unesterified HG distribution in cell wall in the graft union at 20 DAG. Merged images combined the labelling with LM19 and calcofluor counterstaining. *Bars*: **A**–**G** = 100 μm. **H**, **K** = 50 μm. **I** = 500 μm. **J**, **L** = 10 μm. *Abbreviations*: *al,* adhesion line; *cz* cut zone, *DPV* damaged pre-existing vascular tissue, *PV* pre-existing vascular tissue, *RS* rootstock, *SC* scion, *VC* vascular connection. Original source of A–I images: Martínez-Romera et al. (2021)

LM19 immunolabelling (preference for unesterified HG) showed a high increase in the scion and rootstock cut zone after autografting (Fig. 2). During early DAGs, labelling was highly concentrated in the first cell layers of the cut zone (Fig. 2A–D and J, L for details). At 1 DAG, the fluorescence intensity for LM19 immunolabeling in the cut edge of scion (Fig. 2A) was three times higher than that obtained in the innermost tissues (91 ± 9 fluorescence units in the cut edge vs 30 ± 2 in the inner tissues). Similar results were obtained in rootstock (Fig 2B) as a 2.4-fold higher immunofluorescence for LM19 epitope was quantified when the cut edge (94 ± 8 fluorescence units) was compared with inner tissues (39 ± 4 fluorescence units).

LM19 immunolabelling was also observed in the cut area of the injured vascular bundles (Fig. 2D). At 4 DAG, proliferation of callus and vascular cells elements was observed (Fig. 2E, F). At 20 DAG (after graft reconnection), unesterified HG was detected in the adhesion line between scion and rootstock (Fig. 2G, H). Additionally, a more intense labelling was noted collocating with both pre-existing and connecting vasculature (Fig. 2I). The accumulation of fluorescence was strong in the inner side of the cell walls (Fig. 2L). JIM5 labelling (partially esterified and unesterified HG) was faint early after grafting compared with LM20, and showed no clear differences compared to 0 DAG (data not shown), although small patches of intense fluorescence were already observed in some areas of the cut edge (Fig. S2). JIM5 labelling became more intense at 8 and 12 DAG and was present and differentially distributed in the tissue among callus in the graft junction zone at this time points (Fig. S2).

**Fig. 3:**
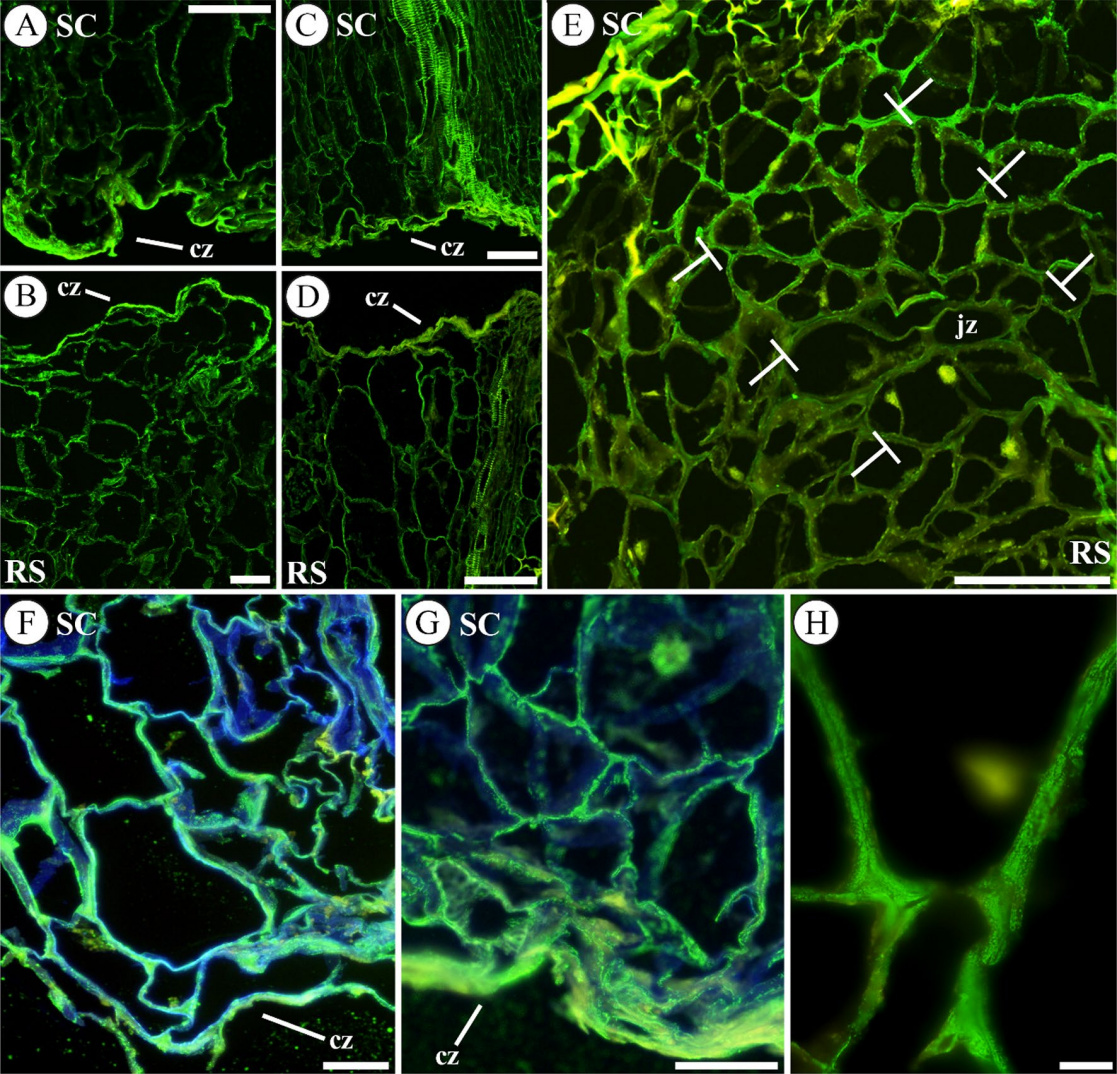
Esterified HG distribution throughout graft union. Representative images of autograft longitudinal sections immunolabelled with LM20 at 1 (**A**, **B**, **F**), 2 (**C**, **D**, **G)** and 20 (**E, H**) days after grafting (DAG). **A**–**D** Note the accumulation of esterified HG in the cut zone (cz). **E** Note the presence of esterified HG in the junction zone (jz) of callus delimited by T lines (┬). **F**, **G** Details of the presence of esterified HG in the cell walls of the cut zone, even forming a matrix too, at 1 and 2 DAG respectively. Observe the labelling in cellular vesicles. **H** Detail of esterified HG in cell walls of the graft union at 20 DAG, note the fluorescence in primary cell walls. Merged images combined the labelling with LM20 and calcofluor counterstaining. *Bars*: **A**–**E** = 100 μm.** F**, **G** = 50 μm. **H** = 10 μm. *Abbreviations*: *cz* cut zone, *jz* junction zone, *RS* rootstock, *SC* scion. Original source of **A**–**E**, **H** images: Martínez-Romera et al. (2021)

Although the labelling for esterified HG (LM20; Fig. 3A–D) was lower than that obtained for unesterified HG (compare with Fig. 2A–D), the immunofluorescence for LM20 epitopes was also observed with high intensity in the cut zone during early graft healing (Fig. 3A–D and F, G for details). As in the case of LM19 labelling, an asymmetry in the distribution of esterified HG between cut edges and inner tissues was observed and quantified. At 1 DAG, fluorescence units of cell wall structures in scion were 92 ± 6 for cut edge compared to 47 ± 5 for in inner tissue. Immunofluorescence quantification in rootstock rendered 74 ± 1 units in cut edge versus 31 ± 4 units in internal tissues (Fig. 3A, B). At 20 DAG esterified HG was also observed in the junction zone, (Fig. 3E) mainly in the primary cell wall of the callus cells (Fig. 3H). Cell wall JIM7 labelling of partially esterified HG, did not change drastically during grafting. Also, it was evenly distributed in the tissues of the graft junction (Fig. S3). On the other hand, protoplasmic labelling of partially esterified HG (probably in vesicles) apparently increased from 1 to 8 DAG and decrease from 8 to 12 DAG (Fig. S3).

**Fig. 4:**
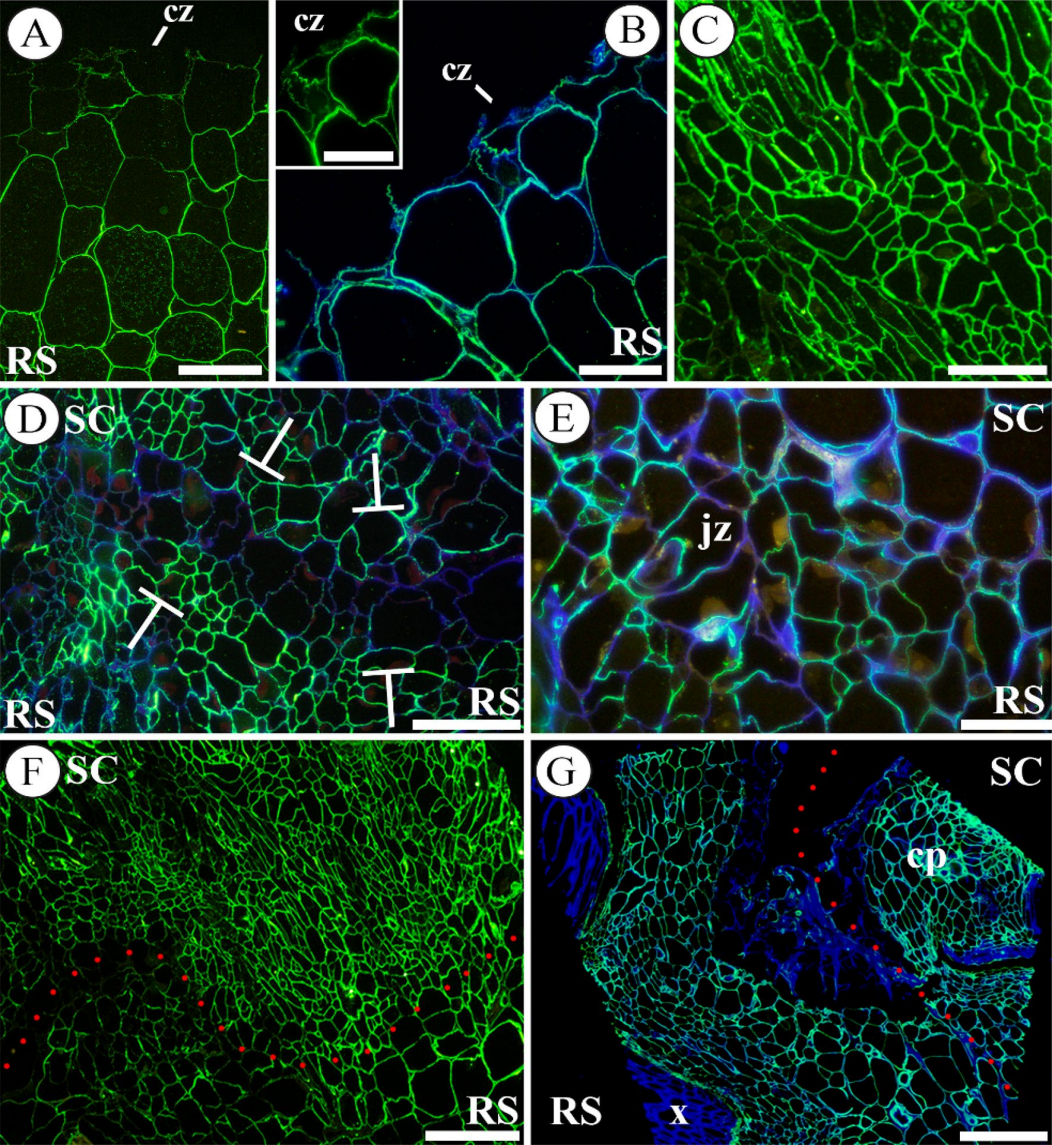
Galactan side chains of RGI immunolocalizated with LM5. Images of representative longitudinal sections from autografts at 1 (**A**), 2 (**B**), 8 (**C**–**F**), and 20 (**G**) days after grafting (DAG) are showed. **A**, **B** Note the distribution of galactan chains in the cut zone (cz) of rootstocks; accumulation of this epitope in the cell walls near the cut was not observed. However, intracellular detection of the epitope was perceived. Insert in **B** is a detail of the cut zone with LM5 labeling only. **C** Callus tissue at 8 DAG, note the different morphology of cells and an increase in galactan chains labelling regard early times. **D** Note that a linear array of cells located at the plane of the graft union (presumably junction zone, delimited by T lines (┬)) shown a low intensity or lack of labelling. **E** Detail of callus junction zone; observe the heterogeneity of galactan chains labelling on the cell walls. **F** Asymmetry of galactan chains labelling was detected between scion and rootstock. Note the difference in the intensity of labelling. Dotted red line indicates the junction zone. **G** Galactan chains labelling did not appear in the xylem and some labelling was present in dead cells and in the cell wall material of the outer layers. Dotted red line indicates the junction zone. **B**, **D**, **E**, **G** Merged images combined the labelling with LM5 and calcofluor counterstaining. *Bars*:** A**, **C**, **D**, **G** = 100 μm; **B**, **E** = 50 μm; **F** = 200 μm. *Abbreviations*: *cp* callus protuberance, *cz* cut zone, *jz* junction zone, *RS* rootstock, *SC* scion, *x* xylem

Regarding the other major pectic polysaccharide, RGI, monitoring during the autograft healing of its galactan and arabinan side chains, by LM5 (Fig. 4) and LM6 (Fig. S4) antibodies, respectively, showed only remarkable changes in the first one. At short times (1–2 DAG) no increase in the accumulation of RGI galactan chains labelling was detected (Fig. 4A, B). However, at 8 DAG the intensity of labelling increased (Fig. 4C). Nevertheless, several cells located at the plane of the graft union showed a lack of labelling (Fig. 4D). Also, an asymmetric distribution of labelling between scion and rootstock was detected at 8 DAG (Fig. 4D–F) as LM5 probed epitopes were more abundant in scion tissues than in rootstock ones.

### Hemicelluloses

**Fig. 5:**
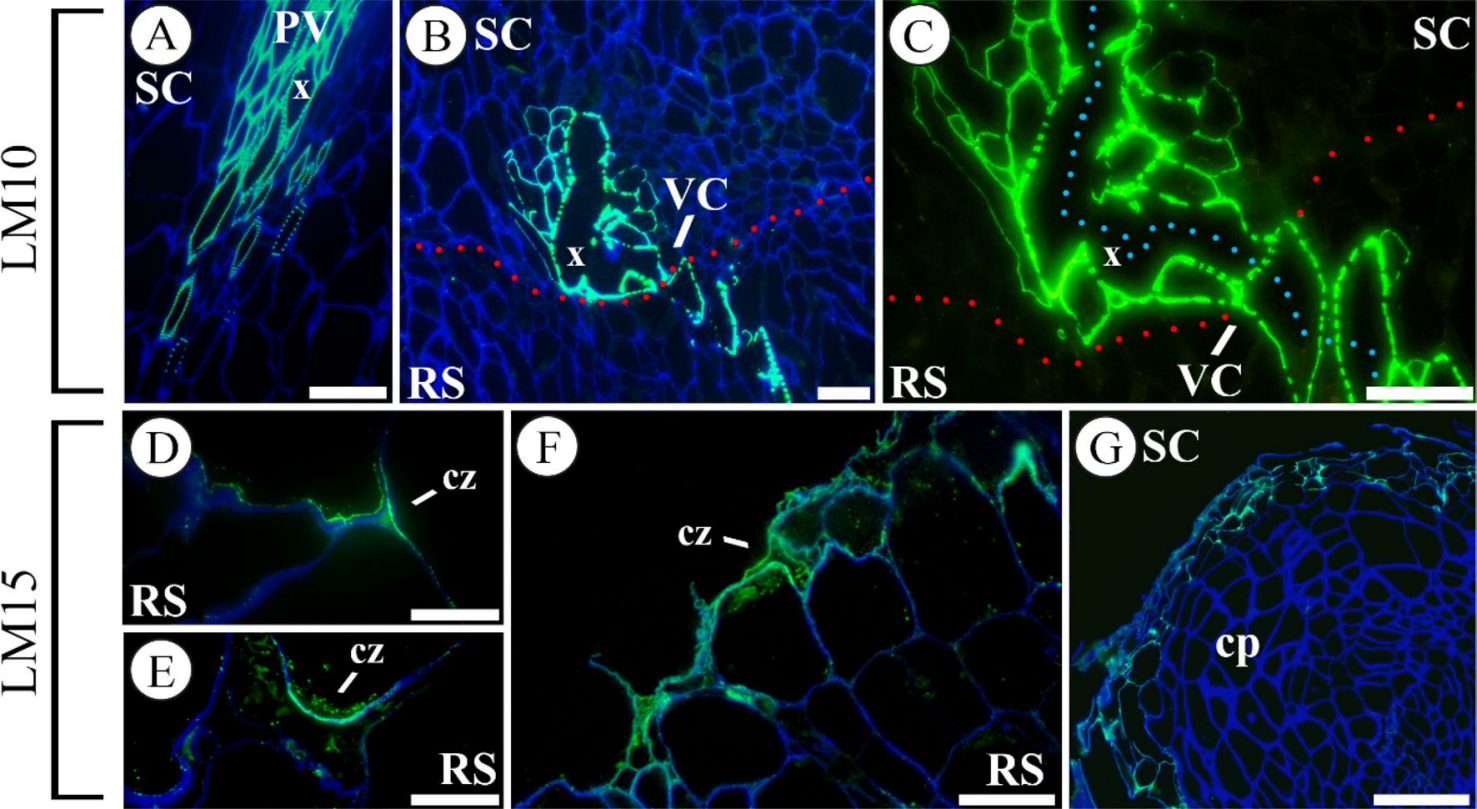
Hemicellulose distribution throughout graft union. Immunolocalization of xylan epitopes, labelled by LM10 (**A**–**C**) and xyloglucan epitopes, labelled by LM15 (**D**–**G**), at 1 (**A**, **D**), 2 (**E**, **F**), 8 (**B**), 10 (**C**) and 20 (**G**) days after grafting (DAG) in longitudinal sections. **A** Scion section. Strong labelling is shown in the xylem conducting cells. **B**, **C** Sections of graft union callus, note a part of the vascular reconnection involving xylem conducting cells. Dotted red line indicates the junction zone and the blue line marks the vasculature continuity. **D**–**F** Sections of rootstocks with high deposition of xyloglucan in the cut zone (cz). **G** Sections of callus protuberance (cp), note the LM15 labelling in the external cells. *All images*, except **C**, are merged images combined the labelling with LM10 or LM15 and calcofluor counterstaining. *Bars*: **A**, **B**, **G** = 100 μm; **C**, **F** = 50 μm; **D**, **E** = 25 μm. *Abbreviations*: *cp* callus protuberance, *cz* cut zone, *PV* pre-existing vascular tissue, *RS* rootstock, *SC* scion, *VC* vascular connection, *x* xylem

The hemicelluloses probed, xylan and xyloglucan, showed a specific distribution along the graft union during autograft healing. The xylan epitope (LM10-probed) was restricted to xylem cells and fibres, and indicated xylem differentiation throughout the autograft healing (Fig. 5A–C). On the other hand, an intense labelling for LM15-probed xyloglucan was detected at the cut edges in the early times after grafting (Fig. 5D–F). At 2 DAG, fluorescence units of cell wall structures of cut edge were 93 ± 10 *vs* 38 ± 2 of inner tissues in rootstock (Fig. 5F). At 20 DAG, xyloglucan was detected in the cell walls of the outer cell layers of the union callus (Fig. 5G).

### Arabinogalactan proteins (AGPs)

**Fig. 6:**
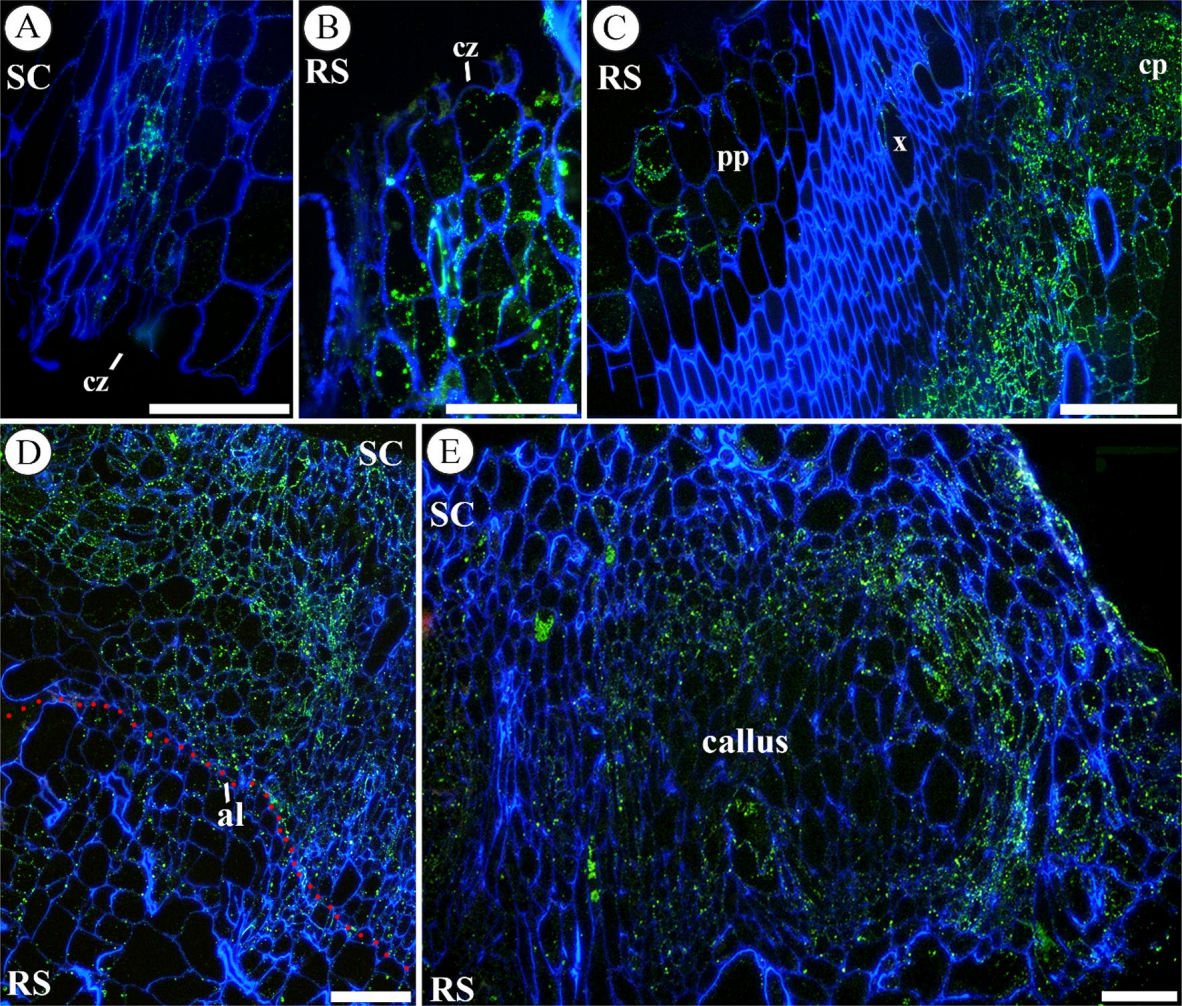
Immunolocalization of AGPs (labelled by LM2), counterstained with calcofluor, throughout the graft union at 1 (**A**), 2 (**B**, **C**), 8 (**D**) and 12 (**E**) days after grafting (DAG) in longitudinal sections are showed. **A**–**C** Sections of scion and rootstock at early times after grafting, note the increase over 1 DAG in AGPs labelling. **D** Section of graft junction, AGPs labelling was strong, and it showed an asymmetrical distribution, being more intense in the scion. **E** Section of a graft junction, note the mature callus tissue with cell areas with different AGP distribution. *Bars*: **A**, **B**, **E** = 100 μm; **C**, **D** = 200 μm. *Abbreviations*: *al* adhesion line, *cp* cortical parenchyma, *cz* cut zone, *pp* pith parenchyma, *RS* rootstock, *SC* scion, *x* xylem

AGPs (LM2-probed) were detected notably associated to the cytoplasm of living cells in vascular bundles. An increase in LM2 labelling was detected during autograft healing evolution (Fig. 6A–C). Moreover, an asymmetric distribution of this epitope was observed: scion-derived tissues showed stronger LM2 labelling than the rootstock tissues (Fig. 6D). In mature callus tissue at 12 DAG LM2 labelling was found only in several areas of cells (Fig. 6E).

## Discussion

Cell wall immunolabelling indicated changes in the abundance and distribution of matrix polysaccharides and AGPs during autograft healing in tomato. Table 2 summarizes these changes.

**Table 2:** Summary of changes in the level and pattern of immunolabelling of matrix cell wall polysaccharides and AGPs during tomato autograft healing

Antibody	Epitope	Trend	Main observations
*Pectins: homogalacturonan (HG) and rhamnogalacturonan I (RGI)*			
JIM5	Partially methyl-esterified and can also bind to unesterified HG	+	Synthesis and deposition, particularly in the cell walls of the cut edges
JIM7	Partially methyl-esterified HG	+	Synthesis and deposition, particularly in the protoplast
LM19	Range of HG samples, preference to unesterified HG	+ + + +	High synthesis and deposition, particularly in the cell walls of the cut edges at early times
LM20	Does not bind to unesterified HG (requires methyl-esters for recognition)	+ + +	Relevant synthesis and deposition, particularly in the cell walls of the cut edges at early times
LM5	Linear tetrasaccharide in (1–4)-β-d-galactans (RGI side chain)	+	Synthesis and deposition, highest from 8 DAG onwards. Some cells of graft union tissue were unlabelled
LM6	Linear pentasaccharide in (1–5)-α-l-arabinans (RGI side chain)	±	No clear changes were detected
*Hemicelluloses*			
LM10	Unsubstituted and relatively low-substituted xylans	±	Associated with secondary cell walls cells: fibres and xylem elements. Useful for monitoring vascular reconnection
LM15	XXXG motif of xyloglucan	+	Synthesis and deposition in the cell walls of cut edges at early times (1–2 DAG)
*Arabinogalactan proteins (AGPs)*			
LM2	AGP protein (carbohydrate epitope containing β-linked glucuronic acid)	+ +	Synthesis and deposition, highest on 8 DAG. Scion – rootstock asymmetry (higher in scion)

*Antibody* means the primary antibody used to detection. *Trend* implies approximately representative pattern after autografting (+ indicates increasing trend; ± equal)

**Fig. 7:**
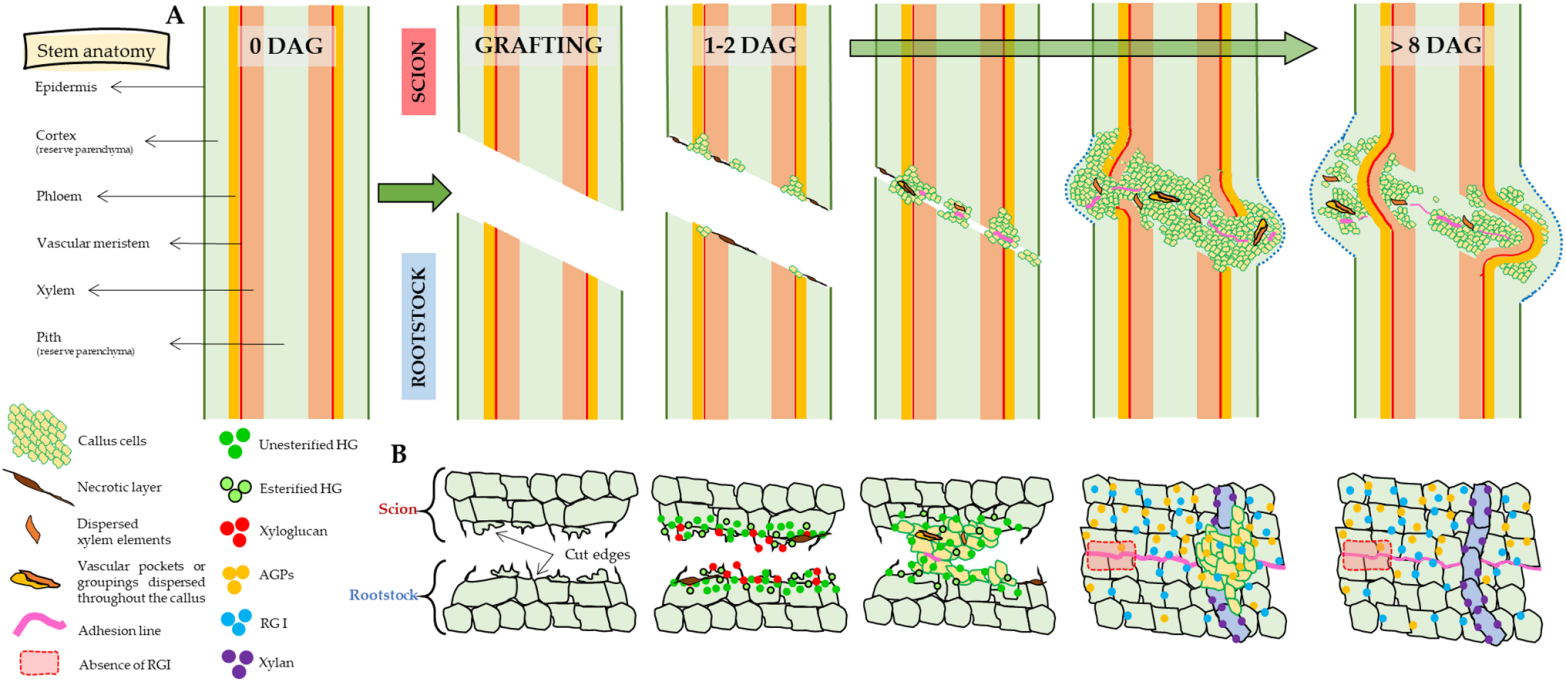
Schematic view of the main events in tissue development during autograft healing (**A**) and summary of the changes in cell wall matrix polysaccharides based in this study (**B**). At early days after grafting (DAG) the accumulation of HG, especially unesterified, and xyloglucan was observed in the cut zone of scion and rootstock. HG epitopes were distributed in callus junction zone during graft healing. Later, an asymmetry between scion and rootstock is observed, consisting of the accumulation of AGP and RGI epitopes in scion tissues. Areas of unlabelled RGI were detected in the graft junction. Finally, xylan epitopes were associated with xylem development and reconnection

The graft healing process is a complex phenomenon involving extensive tissue modifications. Changes in the localization of matrix polysaccharides as well as in the abundance of AGPs are relevant in this context. Figure 7 depicts this process and outlines the epitope labelling location information obtained by IHC in this study.

The adhesion stage (1–4 DAG) is indispensable for the reconstitution of the connection between the scion and the rootstock, and cell walls at the junction zone are expected to have a key role for the adherence of grafted tissues. As it has been previously demonstrated, an immediate consequence of grafting is the secretion of pectins at the junction zone (Jeffree and Yeoman 1983; Yeoman 1993; Sala et al. 2019; Frey et al. 2022); however, the type and distributions of these pectins remain poorly understood. In our experimental system-consisting of tomato autografts-HG epitopes increased on the cut surface at early DAG. This is in consistent with previous findings consisting of the appearance of an extracellular matrix enriched in low methyl-esterified HG in *Arabidopsis* grafts (Sala et al. 2019) and the measured increase of pectins in tissues near the cut edges during tomato graft healing (Frey et al. 2022). Low methyl-esterified HG have adherent properties by its ability to form gels by calcium bridging that may contribute to tissue adhesion (like an “egg box” structure) (Seymour & Knox 2002). In our study, IHC showed that epitopes for low methyl-esterified HG (LM19 labelling) were more abundant than those for medium and high methyl-esterified HG (LM20 labelling). This result indicates that newly synthesized HG would contribute to scion-rootstock adhesion via de-esterification and calcium bridging. Indeed, by cell fractionation, CDTA-extracted pectins increased along tomato graft healing (Frey et al. 2022), consistent with the increase in low methyl-esterified HG found in this work.

Another pectic polysaccharide whose evolution along autografting was evidenced was RGI. By probing its galactan side chains by LM5, a slight increase of RGI abundance was recorded up to 8 DAG, similar to that reported by Frey et al. (2022) by immuno-dot assay of cell wall fractions. This epitope is absent in proliferating meristem cells and present during cell differentiation (Seymour and Knox 2002). Also, the galactan side chains of RGI have been proposed to play a role in the cessation of cell elongation and the onset of secondary cell wall deposition (Seymour and Knox 2002; Moneo-Sánchez et al. 2020; Sun et al. 2021). Additionally, it plays a role in gelation and therefore cell wall adhesion (Mikshina et al. 2017; Kaczmarska et al. 2022), and LM5 labelling has been associated with cell differentiation during in-vitro organogenesis in poplar (García-Angulo et al. 2018). The distribution of LM5 labelling during autografting indicates the absence of RGI galactan chains in some cells in the centre of the callus. Based on the above findings, it would indicate that the centre of the callus has a high cellular proliferating activity and therefore still little cell differentiation activities.

Hemicelluloses showed interesting information about the role of cell wall during autograft healing. Xylan (LM10) labelling revealed that vascular reconnection between scion and rootstock already occurred at 8 DAG. On the other hand, xyloglucan showed a clear increase of labelling limited to cell walls at the cut zone, shortly after autografting. This observation is consistent with the increased LM15 labelling detected very soon after grafting in the close vicinity to cut zone in previous work (Frey et al. 2022). The early presence of xyloglucan epitopes at the cut zone would indicate the former edge of the cell–cell adhesion planes/intercellular spaces. This insight is related to the described association of the LM15 epitope with the edge of the adhesion planes in pericarp cells of unripe tomatoes (Ordaz-Ortiz et al. 2009). Moreover, the labelling pattern of the LM15 epitope was restricted to discrete areas of the cell walls. This regional accumulation of the LM15 epitope might suggest an increase of the hotspot at the local point as a result of cell wall reshaping (Park and Cosgrove 2012, 2015). Following this hypothesis, a certain fraction of xyloglucan is closely interlaced with cellulose chains at discrete sites, forming relatively inaccessible adhesion zones linking two or more microfibrils (Park and Cosgrove 2012, 2015). Therefore, increased detection of the LM15 epitope in these areas would indicate an increase in such biomechanical hotspots, which would be related to a higher cell–cell adhesion at the graft junction. Furthermore, the presence of the epitope early in grafting would imply that these areas undergo active cell wall remodelling during the adhesion phase (Hayashi and Kaida 2011).

Finally, AGPs are structural and highly glycosylated proteins related to developmental processes such as cell–cell recognition, cell differentiation, cell expansion, xylem development, etc. (Ellis et al. 2010; Tan et al. 2012). In this work, AGPs were localised in the cytoplasm of vascular cells, similar to previously reported by Sala et al. (2019). The cytoplasmic localisation of AGP epitopes makes it unlikely that they could play a structural role. Instead, the presence of labelling for AGPs in certain areas of the graft could indicate the occurrence of cells that will actively undergo differentiation (Kreuger and vanHolst 1996). This function would fit well with the increase up to 8 DAG of AGP labelling in internal areas of the graft. An interesting result arising from the labelling pattern for AGPs was the asymmetry in the abundance of AGPs between scion (high) and rootstock (low). Again, this result could reflect concomitant differences in the intensity of cell differentiation processes between both partners.

In summary, the spatiotemporal changes of cell wall components, such as HG, xyloglucan and AGPs, indicate that they have important roles for successful autograft healing, especially in the adhesion phase (early times after grafting). Some of these changes correlate with previous transcriptomic analysis reporting the significant overexpression of genes for cell wall biogenesis and remodelling (such as xylan biosynthetic processes) occurring thorough grafting in tomato plants (Cui et al. 2021; Xie et al. 2022).

This knowledge can be used to develop refined methods to improve graft success. Recently, the application of cellulase (Kawakatsu et al. 2020; Zhang et al. 2021) or pectinase (Zhang et al. 2021) in combination or not with auxins or cytokinins, has been shown to facilitate grafting in *Arabidopsis* by modifying the composition/structure of cell walls from tissues directly involved in the process. These findings open the door to the application of composites containing the major matrix polysaccharides whose changes occur early, as detected in this work, such as low-esterified HG and xyloglucan (alone or as mixtures) in the cutting zone at the time of graft formation to accelerate and/or enhance the grafting process in tomato plants or other species.

In addition, future research addressing the quantification and study of these polysaccharides and AGPs in different types of tomato grafts–such as heterografts with low grafting success–could help in the advance of grafting knowledge and even in plant breeding to obtain better grafted tomato cultivars, and similarly other grafted plants, improving their healing and increasing yield.

### Supplementary Information

Below is the link to the electronic supplementary material.

Supplementary file1 (DOCX 6323 KB)

## Data Availability

The datasets generated and/or analysed during the present study are not available to the public due to the authors’ privacy policy, but are available to the corresponding author upon reasonable request.

## Abbreviations

AGP: Arabinogalactan protein
DAG: Days after grafting
HG: Homogalacturonan
IHC: Immunohistochemistry
RGI: Rhamnogalacturonan I
