# Supplementary material for: Immunohistochemical dynamics of cell wall matrix polymers during tomato autograft healing
Source: Plant Mol Biol. 2023 Apr 20;113(6):353–65. doi: 10.1007/s11103-023-01351-7 (PMC10730687; doi:10.1007/s11103-023-01351-7)
Supplement: Supplementary file 1 — Supplementary file1 (DOCX 6323 KB) [file 11103_2023_1351_MOESM1_ESM.docx]

**Immunohistochemical dynamics of cell wall matrix polymers during tomato graft healing**

Carlos **Frey**, Nerea **Martínez-Romera**, Antonio **Encina***, José L. **Acebes***

Área de Fisiología Vegetal. Departamento de Ingeniería y Ciencias Agrarias. Facultad de Ciencias Biológicas y Ambientales. Universidad de León. Campus Vegazana, 24007, León (Spain).

*****Correspondence: A.E. (a.encina@unileon.es), J.L.A. (jl.acebes@unileon.es) – Campus Vegazana, 24007, León (Spain)

**
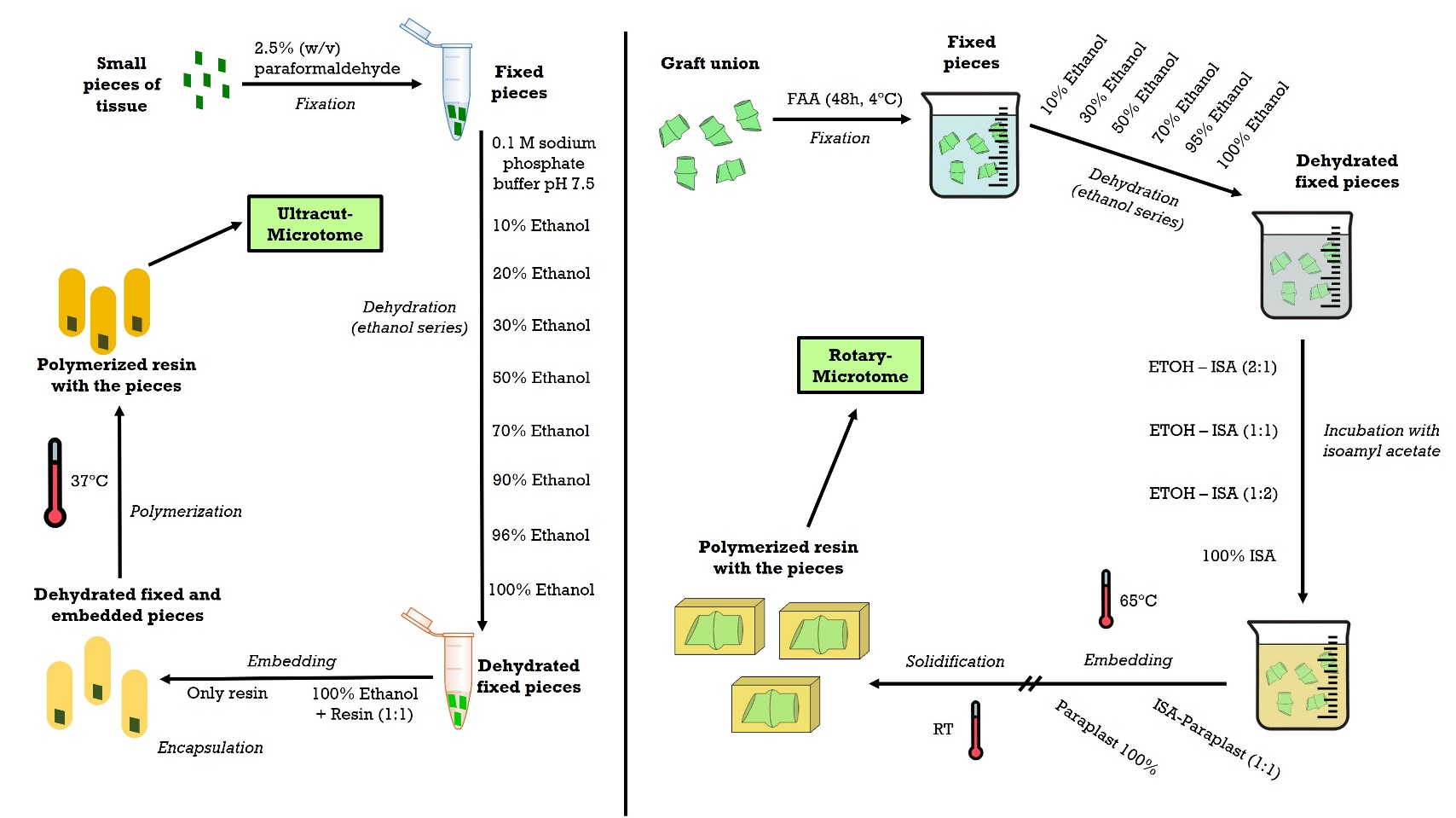
**

**Fig** **S1** Scheme of fixation and imbibition procedures to immunolocalization of cell wall components. **A**. resin protocol (to JIM5; JIM7; LM5; LM6; LM10; LM15; LM1; LM2 antibodies). **B**. paraffin protocol (to LM19 and LM20 antibodies)

**
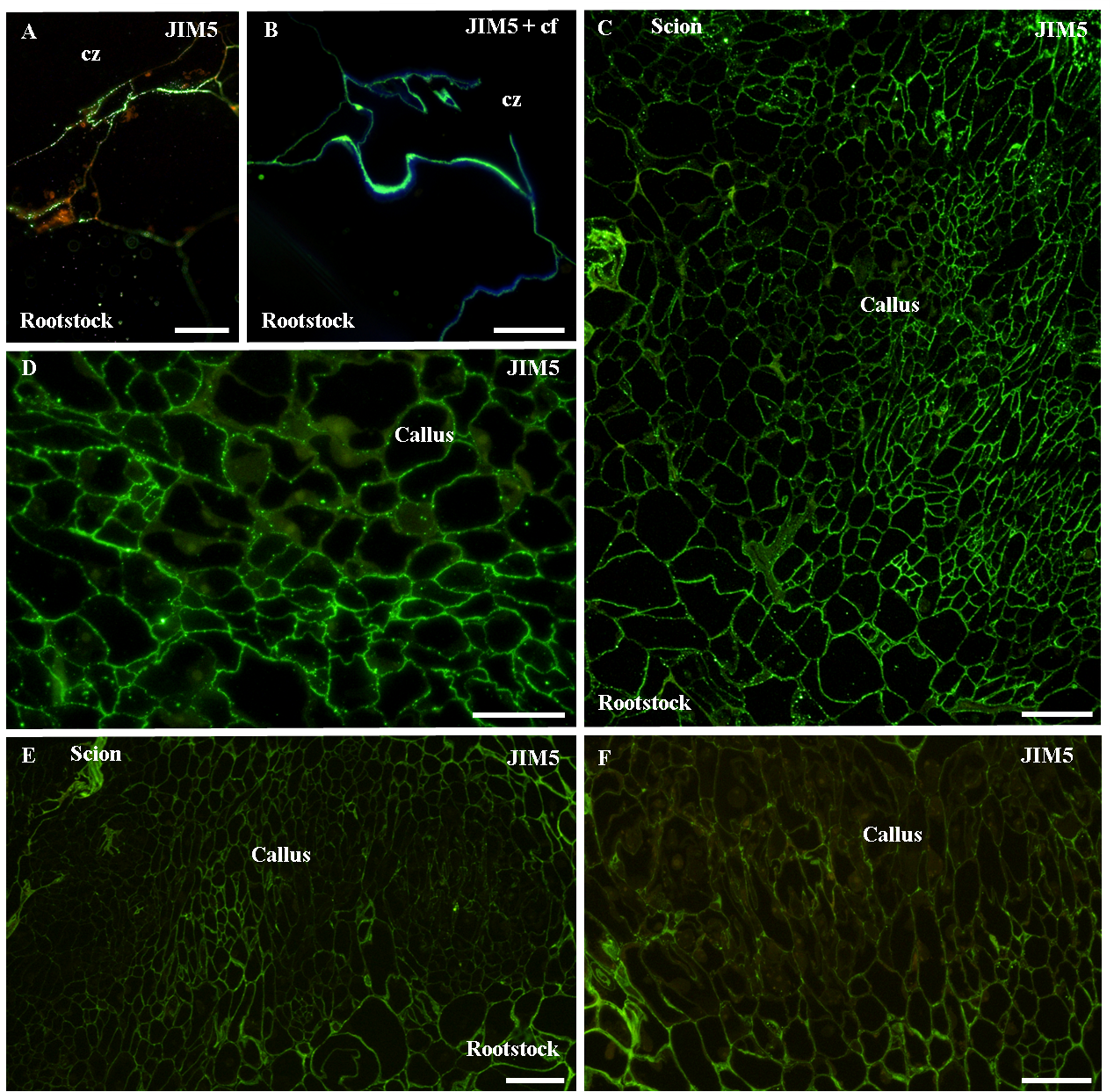
****Fig S2** Graft longitudinal sections labelling with JIM5, that it binds to partially methyl-esterified and unesterified homogalacturonan (green fluorescence). **A**,**B**. Rootstocks partners at 1 day after grafting (DAG), see the green fluorescence accumulation in the walls of the cut zone. **B** is a merged image combining the labelling with JIM5 and Calcofluor counterstaining. **C**. Graft section at 8 DAG, see the JIM5 labelling along the graft union, it is noted an asymmetrical distribution of JIM5 epitope. **D**. Graft junction callus cells at 8 DAG, see the detail of the asymmetric distribution of the epitope, even, some cell walls are partially labelled. **E**,**F**. Graft junction at 12 DAG, see the JIM5 labelling along the graft union, it is noted a differential distribution of the JIM5 epitope too. Fluorescence microscope. Abbreviations: cz, cut zone; cf, calcofluor. Scale bars: **A**,**B** = 25 µm; **C**,**E** = 100 µm; **D**,**F** = 50 µm

**
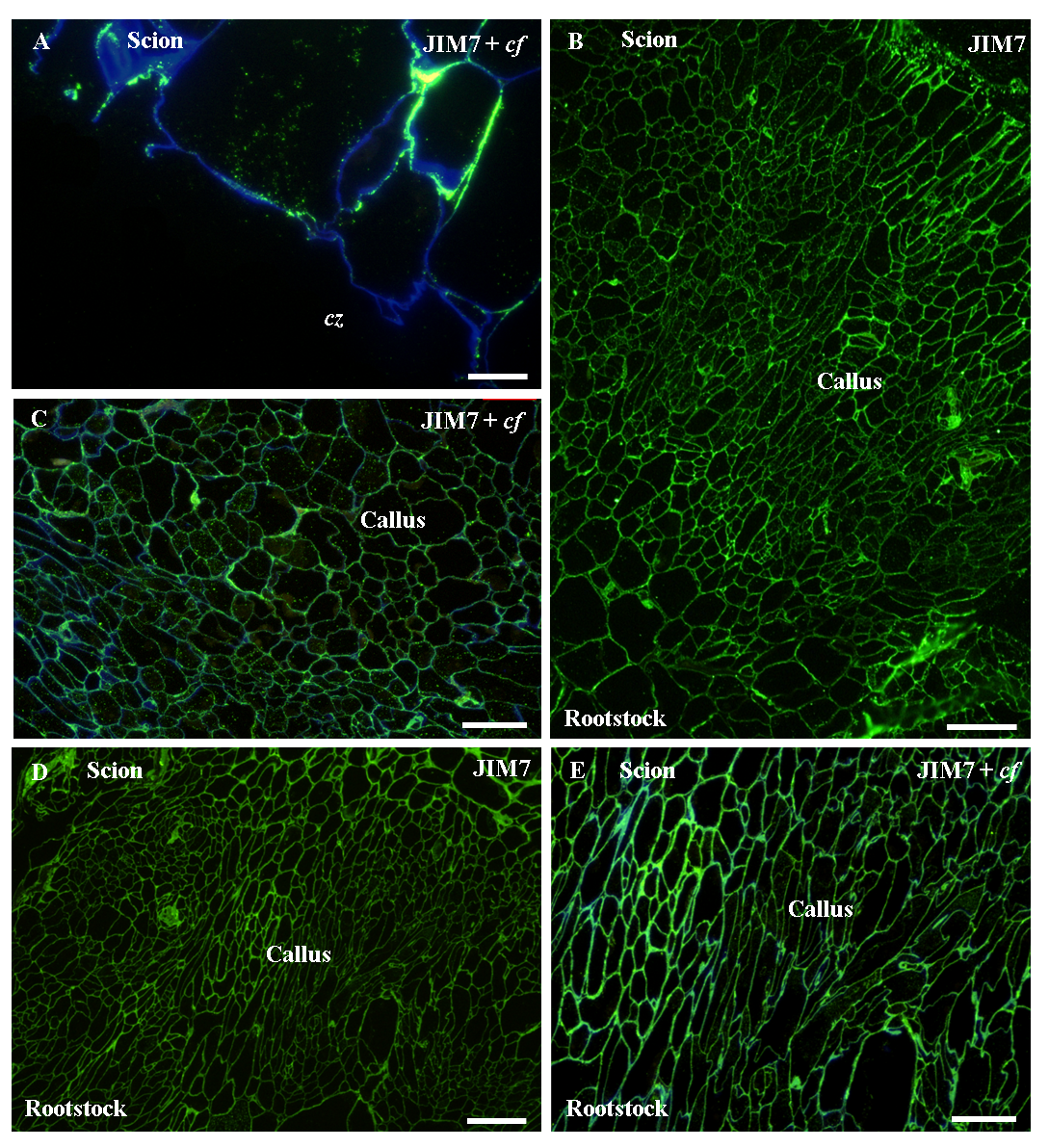
Fig S3** Graft longitudinal sections labelling with JIM7, which binds to partially methyl-esterified homogalacturonan (green fluorescence). **A**. Scion partner at 1 day after grafting (DAG), see the green fluorescence at the cut zone and note the protoplasmic labelling. It is a merged image combining the labelling with JIM7 and Calcofluor counterstaining (blue fluorescence). **B**. Graft junction at 8 DAG, see the JIM7 labelling along the graft union. **C**. Graft junction callus cells at 8 DAG, it is noted the great amount of protoplasmic labelling for JIM7. **D**,**E**. Graft junction at 12 DAG, see the JIM7 labelling along the graft union, it is noted the decreased in the amount of protoplasmic labelling of JIM7 regard 8 DAG. Fluorescence microscope. Abbreviations: cz, cut zone; cf, calcofluor. Scale bars: **A** = 25 µm; **B**,**D** = 100 µm; **C**,**E** = 50 µm

**
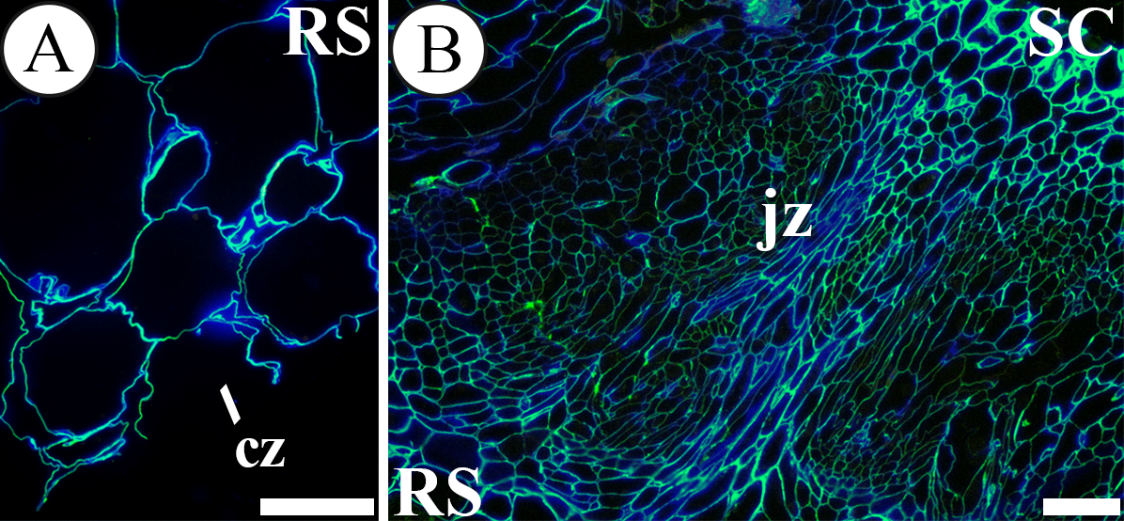
Fig S4** Graft longitudinal sections labelling with LM6, which binds to arabinan side chains of rhamnogalacturonan I (RGI) (green fluorescence), at 1 (**A**) and 12 (**B**) days after grafting. Images are shown merged with Calcofluor counterstaining (blue fluorescence). Clear changes were not detected in the set of images during graft healing. Fluorescence microscope. Abbreviations *cz* cut zone; *jz* junction zone, *RC* rootstock; *SC* scion. Scale bars: **A, B** = 100 μm.
